# Effect of aerobic exercise prior to modified constraint-induced movement therapy outcomes in individuals with chronic hemiparesis: a study protocol for a randomized clinical trial

**DOI:** 10.1186/s12883-019-1421-4

**Published:** 2019-08-15

**Authors:** Erika Shirley Moreira da Silva, Gabriela Lopes Santos, Aparecida Maria Catai, Alexandra Borstad, Natália Pereira Duarte Furtado, Isabela Arruda Verzola Aniceto, Thiago Luiz Russo

**Affiliations:** 1 Department of Physiotherapy, Laboratory of Neurological Physiotherapy Research, Federal University of São Carlos (UFSCar), Rodovia Washington Luís, Km 235, São Carlos, SP 13565-905 Brazil; 2 Health science Institute, Faculty Alfredo Nasse, Aparecida de Goiânia, Goiás, Brazil; 3 Department of Physiotherapy, Cardiovascular Physical Therapy Laboratory, Federal University of São Carlos (UFSCar), São Carlos, SP Brazil; 4 The College of St. Scholastica, EUA, Duluth, Minnesota USA; 5 The Health Unit of the Federal University of São Carlos (UFSCar), São Carlos, SP Brazil

**Keywords:** Stroke, Upper extremity, Motor skill, Aerobic exercise, Motor learning

## Abstract

**Background:**

Recovery of upper limb function in individuals after a stroke remains challenging. Modified constraint-induced movement therapy (m-CIMT) has strong evidence for increasing the use and recovery of sensorimotor function of the paretic upper limb. Recent studies have shown that priming with aerobic exercise prior to task-specific training potentiates upper limb recovery in individuals with stroke. This protocol describes a randomized clinical trial designed to determine whether priming with moderate-high intensity aerobic exercise prior to m-CIMT will improve the manual dexterity of the paretic upper limb in individuals with chronic hemiparesis.

**Methods:**

Sixty-two individuals with chronic hemiparesis will be randomized into two groups: Aerobic exercise + m-CIMT or Stretching + m-CIMT. m-CIMT includes 1) restraint of the nonparetic upper limb for 90% of waking hours, 2) intensive task-oriented training of the paretic upper limb for 3 h/day for 10 days and 3) behavior interventions for improving treatment adherence. Aerobic exercise will be conducted on a stationary bicycle at intervals of moderate to high intensity. Participants will be evaluated at baseline, 3, 30, and 90 days postintervention by the following instruments: Motor Activity Log, Nottingham Sensory Assessment, Wolf Motor Function Test, Box and Block Test, Nine-Hole Peg Test, Stroke Specific Quality of Life Scale and three-dimensional kinematics. The data will be tested for normality and homogeneity. Parametric data will be analyzed by two-way ANOVA with repeated measures and Bonferroni’s adjustment. For nonparametric data, the Friedman test followed by the Wilcoxon test with Bonferroni’s adjustment will be used to compare the ratings for each group. To compare the groups in each assessment, the Mann-Whitney test will be used.

**Discussion:**

This study will provide valuable information about the effect of motor priming for fine upper limb skill improvement in people with chronic poststroke hemiparesis, bringing new evidence about the association of two therapies commonly used in clinical practice.

**Trial registration:**

This trial was retrospectively registered (registration number RBR-83pwm3) on 07 May 2018.

## Background

Stroke is one of the main causes of death and the leading cause of disability in adults worldwide [1–4]. Most poststroke individuals experience a reduction in the function on the affected upper limb [5], related to deficits in force generation, muscular atrophy, joint incoordination, sensitivity disturbances, or spasticity [6]. The reduction in function results in impaired sensorimotor performance during activities of daily living, which may lead to frustration and reinforce compensatory behaviors, such as learned nonuse.

Based on neural plasticity mechanisms [7], Modified Constraint-Induced Movement Therapy (m-CIMT) [8, 9] emerged to provide poststroke individuals with greater functional use of the paretic upper limb [10], reverse learned nonuse [11], and improve motor function and manual dexterity [12]. m-CIMT has high levels of evidence (level A) for the recovery of upper-limb poststroke according to recent guidelines [13, 14]. However, it is not known whether other therapies could prime the effects of m-CIMT.

Recent studies have indicated an enhancement of motor learning (acquisition and retention of motor skills) [15–18], as well as cognitive function (memory, attention, and concentration) [19] in healthy adults when aerobic exercise (AE) was associated with training of specific abilities (motor or cognitive training). According to the literature, the sequence and intensity of AE impact the learning process [20] and might facilitate improvements in motor function or motor memory consolidation processes. The retention of motor tasks improves when performed 15 min after high-intensity interval AE compared to moderate and low-intensity exercise [21, 22]. Learning was greater when AE was achieved using a bicycle compared to a treadmill [23]. A recent study demonstrated that AE on a cycle ergometer, when associated with task-specific training, improves the sensorimotor function of the upper limb [24]. Currently, there is more evidence supporting AE as a method of priming lower limb motor recovery [24, 25]. However, to date, no studies have investigated the effect of AE on fine motor control or relearning of lost upper extremity movement using motion analysis and manual dexterity evaluation [21].

Considering a type of implicit learning, motor priming has been used in neurorehabilitation to facilitate motor learning [22, 26]. The priming theory presumes that when the brain is activated using an intervention delivered prior to the motor learning intervention, it will become more responsive to motor training due to increased neural activity. This “therapeutic window” may result from the modulation of long-term potentiation or long-term depression, such as mechanisms [22, 27, 28]. Movement-based priming, using repetitive or continuous, unilateral or bilateral movements, is an important method of priming the motor cortex in neurorehabilitation [22].

This protocol describes a single-blinded, randomized clinical trial designed to determine whether AE, as a form of movement-based priming, has an effect on the outcomes of m-CIMT, specifically the recovery of the skill of paretic upper limb in persons with chronic stroke. We hypothesize that AE will potentiate m-CIMT, resulting in improved manual dexterity in people in the chronic phase poststroke.

## Methods

This study is a randomized, single-blinded, intention-to-treat controlled clinical trial in which 78 participants of both genders in a chronic stage poststroke will be randomized into two groups of 39 participants. One group will receive AE combined with m-CIMT, and the other group will perform stretching exercises combined with m-CIMT. The eligibility criteria are presented in Table 1.

**Table 1:** Eligibility criteria

Inclusion criteria	
• Stroke resulting in hemiparesis at least six months prior (chronic) • Do not a present lesion in the two hemispheres • Clinical diagnosis of ischemic or hemorrhagic stroke • ≥ 35 and ≤ 80 years old • Participants must present a minimum active movement of 45° of shoulder flexion or abduction, 20° of elbow extension, 10° of wrist extension, 10° of abduction or extension of the thumb, and 10° extension in at least two fingers (metacarpophalangeal and interphalangeal joints) and thumb • Participants must present the ability to pick up a towel using any grip [28] • Participants must present asymmetric of upper extremity, ≤ 2.5 in the Motor Activity Log (MAL) Quantity Scale [10] • Participants must have the ability to remain seated without trunk and arm support for 1 min [29]	
Exclusion criteria	
• Upper limb movement deficits attributable to no stroke pathology	
• Clear signs of dementia or cognitive disorder, indicated in the Mini- Mental State Examination (scores based in years of education, below 13 for illiterate individuals, 18 for individuals with 1 to 7 years of the primary school and 26 for individuals over eight years after primary school) [30, 31] • Individuals with lesion in cerebellar	
• The individuals are smokers, alcohol, or users of illicit drugs at the time of evaluation • Individuals with structural alterations in the cardiovascular and respiratory systems that will contraindicate the aerobic exercise • Individuals with uncorrected auditory and visual deficits • Individuals who present any electrocardiogram alteration or not the cardiorespiratory stress test • Individuals with historical diseases or osteomioarticular alterations • Individuals with body mass index > 28 • Individuals with uncontrolled diabetes mellitus or hypertension, comprehension aphasia, apraxia	

### Sample size

**Fig. 1:**
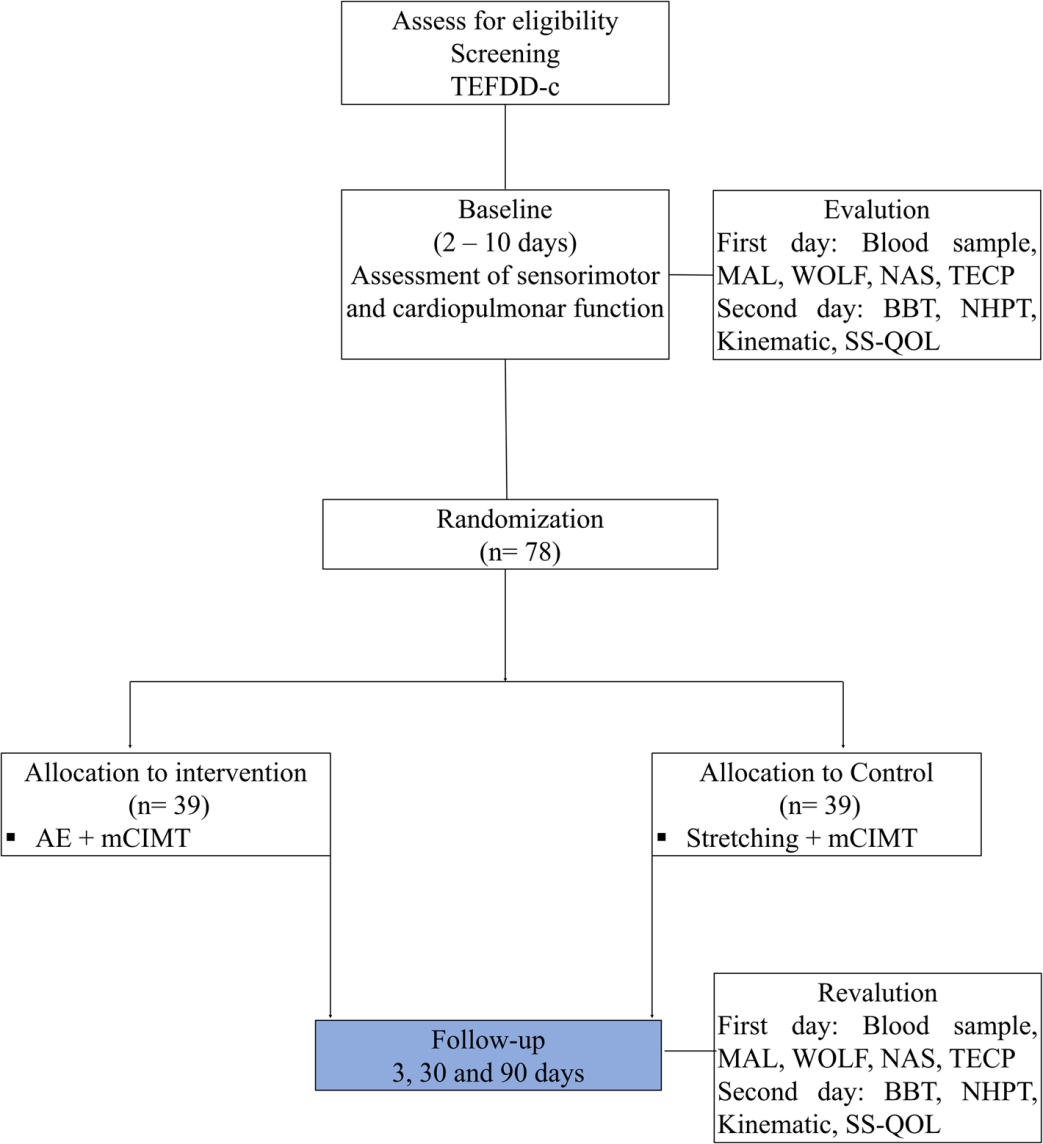
Schematic representation of the experimental design. TEFDD-c: Continuous Dynamic Stress Test; MAL: Motor Activity Log; NAS: Nottingham Assessment Sensory; WMFT: Wolf Motor Function Test; TECP: Cardiopulmonary Exercise Test; BBT: Box and Block Test; NHPT: Nine-Hole Peg Test; SS-QOL: Assessment of quality of life. AE: Aerobic Exercise; m-CIMT: Modified Constraint-Induced Therapy Movement. T1, T2 and T3 will be performed at 3, 30, and 90 days postintervention

The sample size was calculated with G*Power software using the independent t-test, 85% power, alpha 0.05, and 20% drop-out. Data from the Nine-Hole Peg test proposed by Yoon et al. (2014) [29] were considered for this calculation. Thus, a sample size of 78 individuals (39 per group) is required for this study (Fig. 1). However, after collecting the first five participants per group, the sample size will be recalculated for the same variable.

### Randomization and blinding

The randomization method will be matched by age and gender at 1:1 per-muted block randomization generation by a web-based randomization tool (www.random.org). This sequence will be performed independently and remotely by a blinded investigator who will not have contact with other research procedures. Randomization will be concealed until group allocation.

Participants will not be identified by their real names and will not be aware of which study arm they allocated. Thus, the assessors will also be blind, as they will identify the patients by codes and will not have contact with other research procedures. The same standards will be applied to the staff responsible for following the m-CIMT procedures. Data analysis will be conducted by a researcher who is not involved in any stage of recruitment, screening, assessment, or intervention.

### Declarations

This study will be conducted following the principles in the Declaration of Helsinki and approved by the Human Research Ethics Committee at the Federal University of São Carlos (Date: February 2018, Study ID#791060170.0000.5504, UFSCar, Brazil). This study is registered in the Brazilian Clinical Trial Registry (RBR-83pwm3). The authors followed the SPIRIT (Standard Protocol Items: Recommendations for Interventional Trials) guidelines for writing clinical trial protocols [30].

During the consent process, the researcher will clarify the objectives and procedures to be used in the research, including details of the methods to be used, the risks and benefits, and stating the possibility of inclusion in either group. The consent also provides a full guarantee of the freedom of the participant to refuse to participate or withdraw their consent at any stage of the research, without any penalty, and shall maintain the confidentiality and privacy of the participants during all phases of the study. All participants will be asked to provide written informed consent prior to enrollment in the study. In the case an individual is unable to sign, an imprint of the thumb will be requested as evidence of consent. All participants will receive a copy of the consent form approved by the ethics committee.

Data and materials from this clinical trial will be made publicly available at the Federal University of São Carlos (UFSCar). The funding sources for this study are FAPESP (Fundação de Amparo à Pesquisa do Estado de São Paulo; grants: 2017/25185–4 and 2017/13655–6). The authors declare they have no competing interests. The author contributions to this protocol and manuscript are as follows: Study design: ESMS, GLS, AMC, AB, NPDF, IAVA, and TLR. Drafting the manuscript: ESMS and GLS Reviewing and editing the manuscript: ESMS, GLS, AMC, AB, NPDF, IAVA, and TLR.

### Study setting and recruitment

Potential participants will be identified from the health information contained in the medical reports of the School Health Unit and Basic Health Units of the University Hospital, Santa Casa in São Carlos and from the local community using advertisements such as posters and pamphlets.

Potential participants will be screened to determine if they meet eligibility criteria (Table 1), participate in the process of informed consent, and then an initial assessment will be conducted to collect data regarding the sample characterization. The screening, consent and initial assessment will perform in a single day at the individual’s home to ease the burden on participants for transportation. This screening will include a brief medical history and physical examination to verify the eligibility criteria of the individuals. Subsequently, eligible individuals will undergo evaluation of the sensorimotor and cardiopulmonary measures.

Participants will be evaluated on study outcome measures at four time points: baseline, 3 days, 30 days, and 90 days after completion of the protocol. All evaluations will be conducted across two days, with a minimum interval of 24 h and a maximum of 3 days between evaluation days.

Participant screening will include medical history, Mini-Mental Status Examination, physical examination (anthropometric data), active and passive range of motion assessment using goniometer and the questionnaire of the use of the affected upper extremity *Motor Activity Log* (MAL). Moreover, muscle tone assessment by Modified Ashworth Scale [31, 32], upper extremity sensorimotor impairment assessment using Fugl-Meyer Assessment (UE-FMA) [33], Manual preference will be assessed by the Edinburgh Handedness Inventory [34], considering hand preference before the stroke. After screening, if the individuals meet the inclusion criteria, then they will be asked to complete a clinical exercise test conducted by a cardiologist.

The sensorimotor and cardiopulmonary function evaluation will take place in the Physical Therapy Department at UFSCar over two days. On the first day, the assessment of using the affected upper extremity by *Motor Activity Log* (MAL) [35–37], sensory deficit of the upper limb by the Nottingham Assessment Sensory (NAS) [38, 39] and motor function by the *Wolf Motor Function Test* (WMFT) [40, 41] will be carried out, followed by the cardiopulmonary exercise test [42, 43] to determine the training parameters of the AE. On the second day, manual dexterity will be assessed by the *Box and Block Test* (BBT) [44] and the *Nine-Hole Peg Test* (NHPT) [45, 46]. The three-dimensional kinematic analysis will be followed by kinematic analysis of sensorimotor functional activity and the quality of life questionnaire (SS-QOL) [47].

### Primary outcome measures

#### Self-reported upper limb use

Motor Activity Log (MAL) [35–37] is a structured interview that evaluates the spontaneous use of the more affected upper limb. Each item independently asks about how much and how well the most affected upper limb is used in daily activities over the last week. A 6-point amount of use scale (score range, 0–5) quantifies how much the affected arms are used and a 6-point quality of movement scale (score range, 0–5) quantifies how well the affected arms are used [48]. Scores on this measure have adequate reliability and validity in individuals with stroke [35, 36].

#### Manual dexterity

The Box and Block Test (BBT) [44] is a measure of gross manual dexterity consisting of a box divided by a partition into two equal-sized compartments and 100 2.5 cm square wooden blocks. The box will place on the side containing the blocks toward the tested hand. The task is to move the maximum number of wooden blocks, one at a time, across the partition in the middle of the box within 60 s. The hand must crossover the barrier to score one block. Multiple blocks carried over at the same time count as a single block. One practice trial will be performed before the assessment. The score recorded is the number of blocks that were moved for 60 s [44]; the average of three tests will be used to calculate the BBT score. The evaluation will be filmed with a camera placed at a standard position and distance; scoring will be confirmed by evaluation of the video recording.

The Nine-Hole Peg Test (NHPT) [45] assesses fine manual dexterity. The test consists of a nine-peg wooden board with nine holes and a container with nine pegs. The side with the pegs will be placed toward the affected upper limb, and the pegs are picked up and placed in the holes, one at a time, and then removed and placed back in the container [45]. If a peg is dropped, then the examiner quickly places it (or a replacement) in the container. One practice trial will be given before the assessment. The score is recorded as the time in seconds necessary to perform the test [46], and the total score will be calculated by the average of the three trials. The time taken to complete the examination will be recorded with a maximum time of 180 s [46]. If the person does not have enough skill to complete the test, then the pegs/s will be calculated using the number of pegs placed [49] compared to the time limit of 180 s. The evaluation will be filmed with a camera placed at a standard position and distance; scoring will be confirmed by evaluation of the video recording.

### Secondary outcome measures

#### Sensorimotor function

The Nottingham sensory assessment (NSA) [38, 39] will be used to evaluate the sensory deficits (protopathic and epicritic sensory modalities). This assessment has excellent intra- and interrater concordance coefficients and high internal consistency and concurrent validity. There are four subscales: (1) tactile sensation, (2) proprioception, (3) stereognosis, and (4) discrimination between two points on the face, trunk, shoulder, elbow, wrist, hand, knee, ankle, and feet. The tactile sensation subscale evaluates light touch, pressure, pain, tactile location in both limbs and simultaneous bilateral contact. Given that the score on each item ranges from 0 to 2, the total score for the less-affected side ranges from 0 to 90 and the most affected from 0 to 108.

The proprioception subscale evaluates the execution of a movement, its direction, and the articular position of the segments of the more affected half-body. This score varies from 0 to 3, with a total score of 21. The face, trunk, and feet are not evaluated. Stereognosis evaluates the recognition, using the more affected hand, of the following objects: ten centavos, 25 centavos, and one real coin, a pencil, a pen, a comb, scissors, a sponge, a cloth, a glass, and a teacup. The score varies from 0 to 3, with a total score of 21.

The discrimination between points will be tested on the index finger and thenar region; the score varies from 0 to 2, having a total score of 4.

#### Motor function

The Wolf Motor Function Test (WMFT) [40, 41] assesses upper limb functional ability in 17 tasks, measuring the speed of task execution in seconds, the quality of movement through the functional ability scale (FAS), and the strength of grip and shoulder flexion in specific tasks. The execution time total will be quantified by the mean and the median of the FAS scores. The evaluation will be filmed with a camera placed at standard position and distance, and the time and quality of performance will be assigned through analysis of the video.

### Cardiopulmonary testing

Cardiopulmonary exercise testing [42, 43] will be used to determine the training parameters of the AE. Specifically, this test will be used to evaluate the aerobic power and determine the ventilatory anaerobic threshold. The analysis will be performed on an ergometric bicycle (CORIVAL V3, Lode BV, Groningen, Holland). For the test, initially, the participant will stay on the bike resting for one minute. Then, the exercises will start and will be without loads for three minutes; after this, the load increment will start. Power will be calculated for each participant according to the formula described by Wasserman [50]. The increase varies from 10 to 20 watts. Individuals will be instructed to maintain a cadence of 50–60 rpm throughout the test. The test duration will be between 8 and 12 min. The interruption criteria will be as follows: decrease or abnormal heart rate and blood pressure during exercise, presence of arrhythmias, ischemic changes on the electrocardiogram, respiratory distress, dyspnea [42], subjective perception of effort classified as intense [51] or a decrease in cadence below 50 rpm. The metabolic and ventilatory variables, such as pulmonary ventilation (liters\min-1), oxygen consumption and carbon dioxide production (liters\min-1), ratio respiratory exchange, efficiency for oxygen consumption, carbon dioxide and heart rate will be analyzed and captured using an expired gas measurement system (ULTIMA medGraphics-Breeze, St. Paul, Minnesota, USA) and with Breeze Suite 7.1 software (MedGraphics, St. Paul, Minnesota, USA).

### Upper limb performance in 3D kinematics

**Fig. 2:**
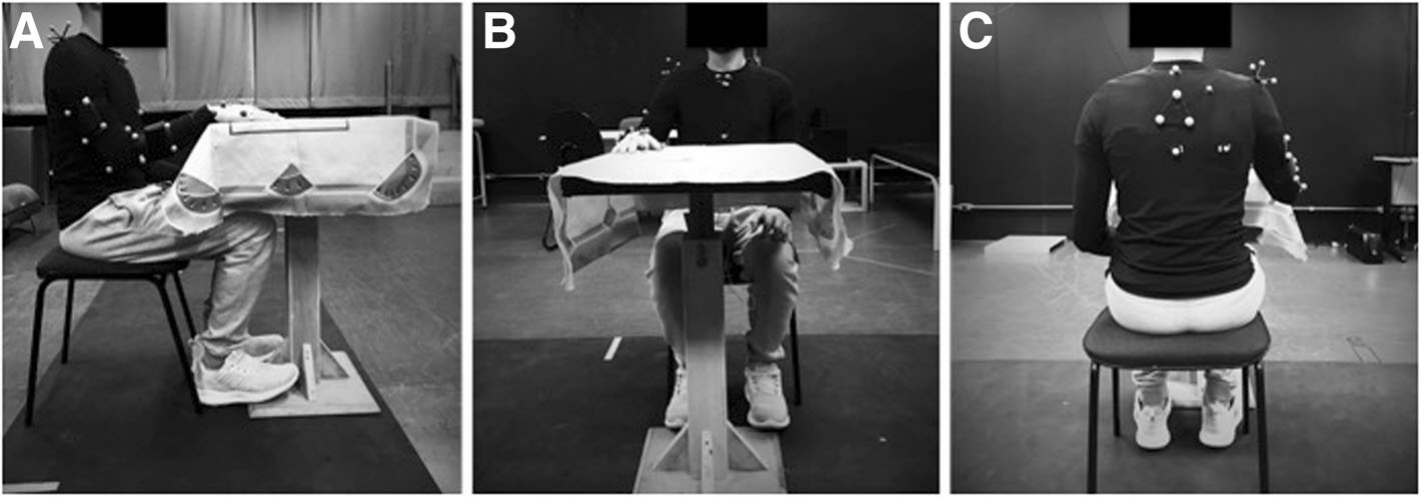
Marker placement for 3D kinematics of the upper limb: A total of 16 markers will be used. Participants will be seated in a chair without back support at an adjustable table at the height of the xiphoid process. **a** Side view: Markers are located on the acromioclavicular joint, lateral and medial epicondyle, midline of the forearm in the direction of the ulnar and radial styloid process. Marker clusters will be placed on the scapula and on the in insertion of the deltoid muscle, 5 cm below the cubital fossa. **b** Front view: Markers will be located on the most ventral point of the sternoclavicular joint, xiphoid process, jugular notch, the base of the proximal phalanx of fingers 2,3 and 5, and the base of the metacarpal of the 3 fingers. Marker clusters will be located on the base of the 3rd metacarpal, the metacarpophalangeal joint of the thumb, digits 2 and 5 and, the tips of the thumb and forefinger and the carpometacarpal joint. **c** Rear view: spinous process C7 and T8, scapula root, lower angle of the scapula. Marker clusters will be located on the thorax between C7 and T8

Three-dimensional Motion Analysis (3DMA) of three functional activities will be quantified using the optoelectronic ProReflex Motion Capture System (Qualisys Medical AB, Gothenburg, Sweden) with eight high-speed cameras at a sampling frequency of 120 Hz. One trained physiotherapist will perform this analysis following the standard protocol of the International Society of Biomechanics (ISB) [52]; (1) placement of clusters on trunk, hemiparetic scapula, arm, forearm, and hand [52–55], (Fig. 2); (2) collection of seated static posture for five seconds; (3) ten passive circumduction movements of the shoulder (right and left sides) to calculate the glenohumeral joint center [56]; (4) removal of anatomical markers; and (5) collection of 3D kinematics during the functional tasks.

**Fig. 3:**
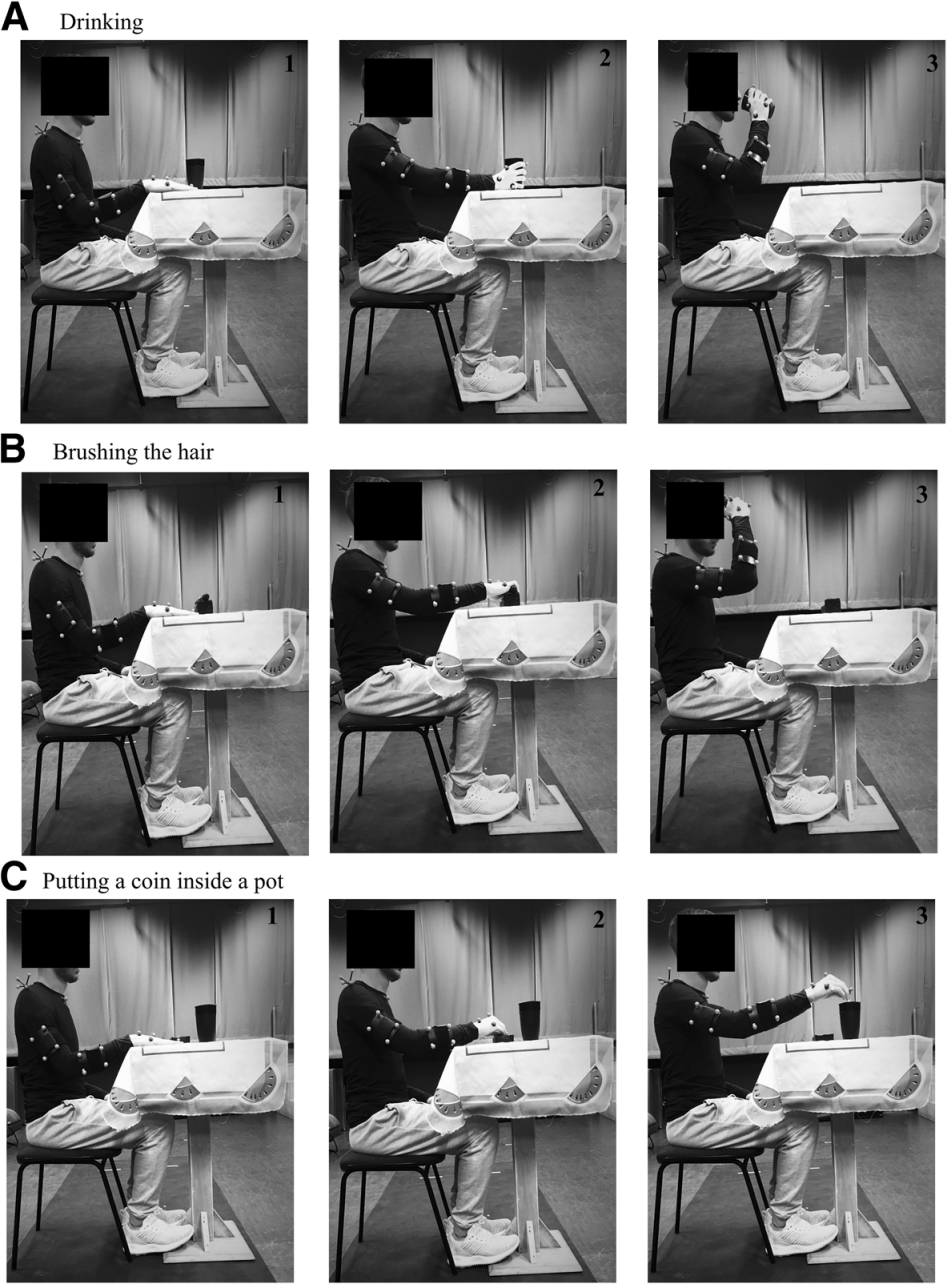
Functional Tasks. **a**. Drinking; **b**. Brushing the hair; **c**. Putting a coin inside a pot. 1. Initial phase; 2. Manipulation phase; 3. Terminal phase

Three functional activities will be evaluated: drinking, brushing the hair, and putting a coin inside a pot (Fig. 3). All objects will be placed on a table in the midline at the height of the xiphoid process and a distance of 80% of the upper limb length, except the pot of the third task, which will place on the same side of the paretic limb at 110% of the upper limb length. The first two tasks will be divided into three phases: (1) reaching for the object, (2) transporting to the body (mouth or head), and (3) returning to the table [57, 58]. The third activity will be divided into two phases: (1) reaching for the coin and (2) transporting to the pot and releasing the coin. All tasks will be performed at a self-selected speed four times with the paretic limb. However, the first trial will be used for familiarization. Thirty-second rest intervals between the trials will be provided.

The UL kinematics calculations will be computed with Upper Limb Evaluation in Motion Analysis software (https://github.com/u0078867/ulema-ul-analyzer) according to ISB recommendations. The following spatiotemporal variables will be calculated for each joint angle per phase: phase duration (second), relative phase duration (ratio between phase duration and total task duration in percentage), peak velocity (mm/s) [53, 54], time to peak velocity, and trajectory deviation (ratio between length of the travelled wrist path and the length of a straight-line connecting start and endpoint) [5, 58–61]. Furthermore, the following will also be calculated: starting angles, range of motion (the difference between the minimum and maximum angle), and joint angles at the point of task achievement (the angle required to complete the task). Hand markers [56] will be used to calculate the maximum aperture between the thumb and second finger, and the time until maximum aperture and the aperture before touching the object will also be calculated.

### Quality of life

The stroke specific of quality of life scale (SS-QOL) [62] assesses of the quality of life in individuals’ poststroke. The instrument has 49 items in 12 subscales (energy, family roles, language, mobility, mood, personality, self-care, social roles, thinking, upper extremity function, vision, and work/productivity). Five response options are offered for each item, with item scores ranging from 1 to 5. Thus, the minimum overall score on the questionnaire is 49 (lowest social participation), and the maximum score is 245 (highest social participation). The SS-QOL has good internal consistency, discriminant validity, criterion validity, concurrent validity, and test-retest reliability [63].

### Intervention protocols

The risk to participants in both intervention groups is minimal and similar to what would be encountered while participating in physical therapy or cardiac rehabilitation. The risks include the feeling of discomfort due to fatigue, the destabilization of vital signs, and frustration due to the difficulty of the tasks. More specifically, participation will be interrupted if they present pain during the procedure, postural hypotension, angina, marked changes in systolic blood pressure (systolic blood pressure ≤ 100 mmHg or > 220 mmHg and diastolic blood pressure < 60 mmHg or > 110 mmHg) or heart rate (maximum heart rate, heart rate max, = 220-age for men or 210-age for women; also, percentage of oxygen < 96% or effort perception > 5 points in the Borg scale (0–10) [64]. When the protocol results in fatigue, frustration or changes in vital signs, the test/training will be paused, and the participant will be further evaluated. If necessary, the participants will be transported to the nearest health unit.

### Aerobic exercise

Aerobic exercise (AE) will be conducted according to the American Heart Association recommendations and based on cardiopulmonary exercise testing [42, 43]. The AE will be performed on a stationary bicycle. During the AE, blood pressure, heart rate, and the perceived effort on the Borg scale–CR10 [51] will be monitored. Measures will be taken every four minutes during training. The AE will be performed at moderate to high-intensity intervals. The AE protocol will involve 10 min of warm-up in a range of 45% HRreserve, 24 min of interval training, followed by 6 min of cooldown between 45 and 60% HRreserve [65–67]. During interval training, every four minutes, the intensities will be alternated between moderate and high (75–90% HRreserve), and the recovery periods will be in moderate intensity (60% HRreserve). In both groups, individuals will receive standardized verbal feedback every 4 min, “Go on, you’re doing well!”. Individuals who do not reach the target moderate and high intensities of HRreserve during training (75–90%) will be encouraged to maintain at least moderate HRreserve intensity (60%). In addition, if the perceived effort limits target HR frequencies, then the participants will be invited to rest for some minutes and return to training until the total time of the protocol is complete. Participants will have a 10-min rest and then begin the m-CIMT protocol. Hydration will be provided to participants throughout the treatment session.

### Control therapy

To equalize participant-to-therapist interaction between groups and reduce the effect on study outcomes, participants in the Stretching + m-CIMT group will also be assisted in performing 40 min of exercise. The exercise will consist of bilateral, intermittent, passive muscle stretching, 3 repetitions with 30-s duration and 60-s intervals between each repetition will be performed for each stretching exercise on the floor or seated. The stretches will be executed for the following muscle groups: hip flexors, knee extensors, ankle flexors, elbow flexors, wrist and fingers flexors.

#### Modified Constraint-Induced Movement Therapy

Modified Constraint-Induced Movement Therapy (m-CIMT) will begin 10 min after the patients have completed the AE or stretching session. The protocol consists of intensive training for 3 h per day for 10 days (two weeks, excluding weekends) and has three components: (1) intensive graded task-oriented training of the paretic upper limb; (2) restriction of the unaffected upper extremity for 90% of the total number of hours awake using a glove; and (3) transfer package, which consists of a behavioral adherence method designed to transfer the gains obtained in the clinical setting to the participants’ real-world environment [15].

Intensive graded task-oriented training of the paretic upper limb includes task training with high repetitions and an increasing level of difficulty between sessions, which vary between patients (shaping). It also involves task training as a whole with a clearly defined context (task practice). The transfer package consists of a list with ten tasks that should be performed at home between each session while wearing the glove and recorded in a home diary. Moreover, in this home diary, individuals must report if they performed the activities at home using the glove and what difficulties they experienced using it. The therapist and the patient choose the tasks in this list at the beginning of the session based on their daily routine. Restraint of the less-affected upper limb will be performed with a removable glove, which allows the limb to be used for support, if necessary, but prevents grasping [40].

In each 3-h session, the use of the glove will be monitored, the MAL will be completed (the first 22 tasks on even days and the last 23 on odd days), and the transfer package will be discussed in the first 30 min. In the remaining hours, upper limb-oriented training of six tasks (six different activities for odd and even days) (Table 2) and one task-practice will be performed. The shaping tasks will vary depending on individual needs and their results in the MAL, which will be individually adjusted (more difficult compared to the previous session) by the therapist. Between each task, a 30-s rest will be given. Whole task practice will be performed to promote the increased motor function of the affected limb during functional activities.

**Table 2:** Table of the tasks performed in shaping

Task	Materials used	Description	Emphasized Movements
Odd days			
1. 1. Put blocks on top of the box	A box and various blocks	The subject moves blocks of wood of the table into the box. The placement and height of the box depend on the desired movement.	Pinch, wrist extension, elbow extension, shoulder flexion
2. Clean the table	A flannel, ruler, scotch tape	The subject is asked to use the flannel, while doing movements of cleaning the table, at a given target.	Elbow extension, shoulder flexion, abduction or adduction depending on the placement of the target.
2. 3. Velcro parts	A checkerboard and the lady’s pieces with Velcro	The checkerboard is used with Velcro in separated houses. The lady’s pieces are of wood with Velcro below the top. The subject is asked to grasp clamp or finger extending and moving the piece from one house to another in the frame.	Pinch or isolated finger movements, wrist flexion/extension, elbow extension, shoulder flexion.
3. 4. Poker chips	A modeling clay and poker chips	The subject is asked the grasp the poker chips, one at a time and put them in the glass. The poker chips can be arranged on the darts board or on a clay mound.	Pinch, wrist extension, elbow extension and shoulder flexion.
4. 5. Open and close the door		The subject is asked to stay front of the door. The distance from the door is measured and marked. The subject practices opening and closing the closet door.	Grip, supination, extension and flexion elbow, flexion and extension shoulder.
5. 6. Placing balls	Tennis, golf ping pong balls and cup	Balls are placing in table and the subject is asked to grasp one at the time and placing in cup.	Various types of grip, elbow extension, shoulder flexion, horizontal adduction/abduction of shoulder.
6. 7. Hockey puck	A hockey puck and scotch tape	Draw a line or put a scotch tape on the table. The subject holds the *hockey puck* and pushes it to the line or to the line drawn trough the elbow extension.	Pinch, elbow extension, abduction shoulder, scapular abduction and protraction.
Even days			
7. 1. Pegboard	A pegboard	The subject raises the wooden stick and places it on a pegboard hole designed. The pegboard can be placed on top of a box to work the shoulder flexion.	Pinch, wrist extension, elbow extension, shoulder flexion.
8. 2. Pasta Roll	A pasta roll	A mark is made on the backing surface of the pasta roll and this is positioned on the table. The participant is asked to handle the pasta roll.	Pinch, ulnar and radial deviation.
9. 3. Turn dominoes	A domino	The dominoes are positioned in front of the subject. The participant is requiring to reach the pieces and turn them.	Pinch or isolated finger movements, wrist extension, supination and pronation of the forearm, shoulder flexion.
10. 4. Fork and meat	Fork, dish and modeling clay	The subject is asked when used the fork to grasp pieces modeling clay and moved to dish, one at a time. The therapist can watch by pulling the pieces of clay from the fork if necessary, but the subject should be encouraged to pull the fork while the therapist holds the clay.	Pinch, elbow extension, shoulder flexion, addition / abduction.
11. 5. Hoop horizontal	Bar and rings	The subject is required to place the rings in a horizontal position on the bar.	Pinch, wrist extension, elbow extension, shoulder flexion, horizontal abduction and adduction.
12. 6. Serve on a mug	A mug with handle, beans or marble, and a cup or bowl.	Beans or marble are placed in a mug. The subject is required to grasp the mug by the handle and pour the beans into the cup or bowl without knocking down any beans, and then put the mug back on the table.	Types of grip, wrist extension, forearm supination/pronation, elbow extension and should flexion.
13. 7. Bottle of water	Bottle of water	The subject is asked to use a cylindrical grip and moving the bottle from one target to another while keeping the forearm in a neutral position.	Cylindrical grip, supination, extension and flexion elbow, flexion and extension shoulder.

### Attrition and adherence

Participants will be withdrawn from the study under the following conditions: a) two consecutive or three alternating absences during treatment sessions; b) inability to complete the posttest and follow-up; or c) development of any disabling condition that precludes participation in the study. Regarding adherence strategies, up to two nonconsecutive absences can be compensated the following week. There will also be flexible hours offered for receiving therapy, as well as direct contact by telephone with participants confirming the evaluation dates and supporting treatment adherence. Additional measures to avoid individuals dropping out are periodic evaluations (during the outcome analyses) on the satisfaction level of the therapy, discussing difficulties in continuing treatment (for example, transport logistics to the laboratory), and attempts to resolve and prevent possible problems that may interfere with adherence and continued participation in the study. Semistructured interviews will be held with each participant.

After the initial five days of participation and following completion of the ten days of therapy. A series of open-ended questions will be asked regarding their impressions, satisfaction, and physical tolerance of the treatment.

### Statistical analysis

Demographic characteristics, such as weight, height and body mass index, will be presented as the mean and standard deviation. Median, maximum and minimum values will be used to describe poststroke time and UE-FMA score.

For all dependent variables, normality (Kolmogorov-Smirnov) and homogeneity (Levene) tests will be applied. If the variables present a normal and homogeneous distribution, then two-way analysis of variance with repeated measurements and Bonferroni’s correction will be used to examine the effect of group-by-evaluation time interaction, group (AE + m-CIMT and Stretching + m-CIMT), and evaluation time (baseline, 3, 30 and 90 days after the intervention). To protect against Type I error, Bonferroni’s correction will be used. Thus, each of the 10 planned comparisons will have to achieve *p* = 0.005 for statistical significance. The partial eta squared (η^2^) will be used to determine the effect size of the intervention. The mean difference from pre- and post-interventions and the corresponding 95% confidence interval (95% CI) will be calculated for each group (EA + m-CIMT and Stretching + m-CIMT) in order to estimate the effect of the intervention.

Otherwise, the Friedman test (*p* < 0.05) will be used followed by the Wilcoxon test with Bonferroni’s adjustment (*p* < 0.008) to compare evaluation time for each group baseline, 3 days, 30 days, 90 days after the end of treatment. The Mann-Whitney test with Bonferroni’s adjustment (*p* < 0.012) will be used to compare the groups in each time evaluation (EA + m-CIMT and Stretching + m-CIMT).

## Discussion

One of the most significant difficulties in neurorehabilitation is the recovery of fine upper limb skills [68] after stroke, and most studies did not evaluate manual dexterity and upper limb performance using 3DMA. Aerobic exercise potentiates neuroplasticity and may improve motor recovery after stroke [19, 63]. Performed alone, aerobic exercise can enhance motor function after stroke; motor learning in stroke rehabilitation may improve when aerobic exercise is performed before motor training. This study will investigate, using a randomized clinical trial, whether AE can enhance the effect of m-CIMT on the manual dexterity recovery in the paretic upper limb of people in the chronic phase poststroke.

According to the literature, the deficit in the UE can negatively affect the quality of life of the survivor; that deficit includes voluntary impairment control of finger extension from coactivation and decreased gross and fine manual dexterity. Poor dexterity is associated with the correlation between the ability to use hands and manipulating objects and independence in life activity.

Kinematic analysis can provide objective, quantitative, accuracy measures of arm motor impairment after stroke, with the ability to detect and quantify differences in movement patterns. Furthermore, these data enable the evaluation of the nature of the functional improvement, namely, the determination of whether compensatory strategies or recovery of normal movement are the cause.

This study will provide valuable information about the effect of motor priming for fine upper limb skill improvement in people with chronic poststroke hemiparesis, generating new evidence about the association of therapies highly used on clinical practice.

## Data Availability

Not applicable.

## Abbreviations

AE: Aerobic exercise
BBT: Box and Block Test
FAS: Functional ability scale
HR_reserve_: Reserve heart rate
MAL: Motor Activity Log
m-CIMT: Modified Constraint-Induced Movement Therapy
NAS: Nottingham Assessment Sensory
NHPT: Nine-Hole Peg Test
SS-QOL: Quality of Life Questionnaire
TEFDD-c: Continuous Dynamic Stress Test
UE-FMA: Upper-Limb Fugl-Meyer Assessment
WMFT: Wolf Motor Function Test
