# Correction to: Effect of aerobic exercise prior to modified constraint-induced movement therapy outcomes in individuals with chronic hemiparesis: a study protocol for a randomized clinical trial

**DOI:** 10.1186/s12883-019-1454-8

**Published:** 2019-09-13

**Authors:** Erika Shirley Moreira da Silva, Gabriela Lopes Santos, Aparecida Maria Catai, Alexandra Borstad, Natália Pereira Duarte Furtado, Isabela Arruda Verzola Aniceto, Thiago Luiz Russo

**Affiliations:** 1 Department of Physiotherapy, Laboratory of Neurological Physiotherapy Research, Federal University of São Carlos (UFSCar), Rodovia Washington Luís, Km 235, São Carlos, SP 13565-905 Brazil; 2 Health Science Institute, Faculty Alfredo Nasse, Aparecida de Goiânia, Goiás, Brazil; 3 Department of Physiotherapy, Cardiovascular Physical Therapy Laboratory, Federal University of São Carlos (UFSCar), São Carlos, SP Brazil; 4 The College of St. Scholastica, EUA, Duluth, MN USA; 5 The Health Unit of the Federal University of São Carlos (UFSCar), São Carlos, SP Brazil

**Correction to: BMC Neurology (2019) 19:196**

**https://doi.org/10.1186/s12883-019-1421-4**

Following publication of the original article [1], the authors reported an error in Table 2 wherein the item numbering in the first column is wrong.

The publishers apologise for this error. The original article [1] has been corrected.
